# Oxidative Stress Markers and the Retinopathy of Prematurity

**DOI:** 10.3390/jcm9092711

**Published:** 2020-08-21

**Authors:** Alessandro Graziosi, Marika Perrotta, Daniele Russo, Giorgia Gasparroni, Claudia D’Egidio, Benedetta Marinelli, Guido Di Marzio, Gennaro Falconio, Leonardo Mastropasqua, Giovanni Li Volti, Rocco Mangifesta, Diego Gazzolo

**Affiliations:** 1 Neonatal Intensive Unit Care, University “G. d’Annunzio” Chieti-Pescara, 66100 Chieti, Italy; a.graziosi@hotmail.it (A.G.); perrottamarika@hotmail.it (M.P.); danielerusso1607@gmail.com (D.R.); giorgia.gasparroni@gmail.com (G.G.); claudia.degidio@hotmail.it (C.D.); 2 Department of Paediatrics, University “G. d’ Annunzio” Chieti-Pescara, 66100 Chieti, Italy; 3 Italian Academy of Traditional Osteopathy (AIOT), 65100 Pescara, Italy; benedettamarinelli.osteo@gmail.com; 4 Department of Ophthalmology, University “G. D’ Annunzio” Chieti-Pescara, 66100 Chieti, Italy; dimarzio61@alice.it (G.D.M.); gennarofalconio@libero.it (G.F.); mastropa@unich.it (L.M.); 5 Department of Biomedical and Biotechnological Sciences, University of Catania, 95100 Catania, Italy; livolti@unict.it; 6 Health and Safety Manager, ASL02 Abruzzo, 66100 Chieti, Italy; rocco.mangifesta@asl2abruzzo.it

**Keywords:** ROP, oxidative stress, antioxidants, biomarkers, lutein

## Abstract

Retinopathy of prematurity (ROP) is a leading cause of potentially preventable blindness in low birth weight preterm infants. Several perinatal and postnatal factors contribute to the incomplete maturation of retinal vascularization, leading to oxidative stress damage. Literature data suggest that the lack of equilibrium between pro-oxidants and anti-oxidants plays a key role. In the last decade, there has been an increasing interest in identifying the antecedents of ROP and the relevant pathogenic mechanisms involved. In this context, a panel of biomarkers was investigated in order to achieve early detection of oxidative stress occurrence and to prevent retinal damage. Several nutritional elements have been found to play a relevant role in ROP prevention. At this stage, no conclusive data have been shown to support the usefulness of one biomarker over another. Recently, the Food and Drugs Administration, the European Medicine Agency, and the National Institute of Health proposed a series of criteria in order to promote the inclusion of new biomarkers in perinatal clinical guidelines and daily practice. The aim of the present review is to offer an update on a panel of biomarkers, currently investigated as potential predictors of ROP, highlighting their strengths and weaknesses.

## 1. Introduction

Retinopathy of prematurity (ROP) is a pathology affecting the retina bloodstream, more frequently found in low birth weight preterm infants (LBWs). Despite improvements in neonatal care and therapeutic strategies, it remains the second cause of childhood blindness after impaired cortical vision. ROP also causes early and late visual sequelae, often associated with neurological developmental disabilities, with a high social impact [1,2,3,4,5,6,7].

Currently, there is a general consensus on the inverse correlation among the incidence of ROP gestational age (GA) and weight (BW) at birth. Additional risk elements are, to date, a matter of debate and investigation, which regard the following factors: maternal prenatal and perinatal social conditions, medical interventions, and nutritional and genetic factors [1,2,3,4,8,9,10,11].

ROP is a multiphasic pathological process linked to the incomplete maturation of retinal vascularization in LBWs. Pathological features of ROP are the onset of retinal ischemia, resulting in aberrant angiogenesis typically associated with fibrovascular proliferation, which may ultimately lead to progressive vitreoretinal traction. In this light, endothelial components such as vascular endothelial growth factor (VEGF) are mainly involved in the cascade of events leading to pathological angiogenesis [12].

Although ROP can occur in about 66% of LBWs, stages I–III of the disease can spontaneously regress at any time. Conversely, stages IV-V, typical of a progression of the disease, infants are at increased risk for vitreous hemorrhage, tractional retinal detachment, and blindness [11,12,13].

Among different and various exogenous factors involved in the genesis of ROP, perinatal therapeutic strategies performed in the delivery room (DR) and in neonatal intensive care units (NICUs) have to be taken into due account. This is especially true for oxygen by means of invasive or non-invasive mechanical respiratory strategies. In fact, oxygen support, under different conditions, has been related to oxidative stress, inflammation, and nutritional capacity, activating angiogenic and neuroprotective growth factors and hypoxia-mediated oxidative species [1,2,3,4,5,6,7,8].

Therefore, the aim of the present review is to offer an update on well-established oxidative stress biomarkers and new potential antioxidant agents in infants complicated by ROP.

## 2. Epidemiology and Risk Factors

Epidemiological studies conducted in the USA have reported that ROP occurs annually in about 14,000 LBWs. In this regard, the Cooperative Cryotherapy for Prematurity Retinopathy group reported ROP incidences in 66% of LBWs weighing ≤ 1.250 g and 82% in those of BW < 1.000 g. Spontaneous regression occurred in about 90% of the cases, whilst medical treatment was necessary for about 1.100–1.500 ROP severe forms. Finally, despite appropriate and prompt treatment, partial or complete blindness accounts for 400–600 LBWs per year [10,11].

### 2.1. Prenatal and Perinatal Factors

GA and BW are the two best-known risk factors correlated with the development of ROP [1,2,3,4,7,8]. In fact, LBWs are more often exposed to supplementation of high concentrations of oxygen for long periods, as well as to continuous changes in oxygen saturation during mechanical ventilation, which constitutes the most identified risk factor for severe ROP. Although several randomized controlled trials have been conducted to identify the optimal oxygen level and saturation that ensure a reduced risk of developing ROP, to date, these ranges have not yet been conclusively elucidated [1,2,3,4,12].

### 2.2. Maternal Factors

Pregnancy-induced hypertension (PIH) is known to be associated with higher levels of antiangiogenic factors such as sFlt-1 (fms-like soluble tyrosine kinase-1), an antagonist of VEGF and placental growth factor. Several studies have found that PIH, including preeclampsia–eclampsia, is a significant risk factor for ROP [1,2,3]. Notably, there are still conflicting results on the association between gestational maternal diabetes (GMD) and ROP. In fact, GMD can cause both the direct (i.e., an increase in retinal VEGF due to hyperglycemia) and indirect (i.e., association with respiratory distress syndrome) development of ROP [1,2,3,4,5].

### 2.3. Postnatal Factors

Racial differences in ROP and a high concordance rate between monozygotic twins strongly suggest a genetic predisposition to ROP, but, to date, no specific genetic variants have been identified. Other risk factors, whose role in the genesis of ROP is still highly debated, are sepsis, blood transfusions, erythropoietin and nitric oxide administration, and intraventricular hemorrhage [1,2,3,13].

## 3. ROP Stages

The classification of ROP consists of 5 stages based on severity and 3 zones describing the area of retina affected by the disease. In Stage 1, a line between vascular and avascular retina sections is visible, followed by a ridge of tissue, where small tufts of neovascular tissue can be found (Stage 2). During the disease’s evolvement, Stage 3 develops with abnormal vessel proliferation along the ridge and into the vitreous chamber. Stage 4 can be divided into 4a and 4b, respectively, characterized by extrafoveal (a) and intrafoveal retinal detachment (b). Finally, in Stage 5, the retina is completely disconnected [7].

## 4. Pathophysiology

The pathogenesis of ROP is characterized by multifactorial agents and complex molecular mechanisms [1,2,3,4,12,13,14,15].

The physiologic human retinal vascularization begins, approximately, at 16 GA, with an evolution from central to periphery at a rate of 0.1 mm/die. Moreover, the two segments of the retina are vascularized at different times during the pregnancy: the nasal retina around 36 GA, while the temporal part around 40 GA.

**Figure 1:**
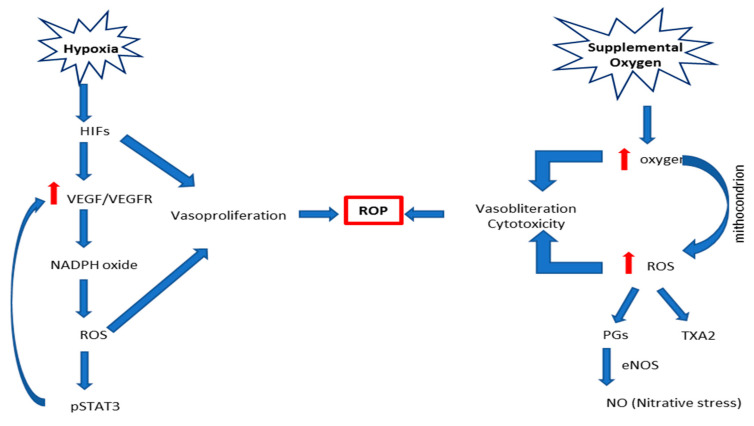
Description of the main known biochemical cascade of events leading to retinopathy of prematurity (ROP). Abbreviations: eNOS, endothelial NO synthase; HIFs, hypoxia-inducible factors; NADPH, dihydronicotinamide-adenine dinucleotide phosphate; NO, nitric oxide; pSTAT3, phosphorylated signal transducer and activator of transcription 3; PGs, prostaglandins; ROP, retinopathy of prematurity; TXA2, thromboxane A2; VEGFR, vascular endothelial growth factor receptor.

The development of ROP advances in two phases [12,13,14,15], at first characterized by (i) retinal vascular growth interruption due to premature birth [12], (ii) bleeding vulnerability to injury and obliteration by any stressor factors such as the amount of oxygen supplementation [12,13,14,15,16], (iii) suppression of vasoproliferation for reduced VEGF and poor cytoprotective molecules [12,13,14,15,16,17], and (iv) LBWs’ need of higher oxygen tension after birth than that in utero, which causes a downregulation of the major hypoxia-triggered VEGF by determining the vaso-obliteration of retinal capillaries [12,13,14,15,16,17]. Moreover, ROP’s second phase consists of (i) retinal vasoproliferation induced by hypoxia and an increased metabolic requirement [12,16], (ii) greater production of hormones (IGF-1) and growth factors (VEGF) to improve retinal perfusion in response to hypoxia [15,16,17], (iii) production of adhesive fibrins influenced by proteins of the extracellular matrix (vitronectin, fibronectin, and fibrinogen) [12,17], and (iv) induction of cellular processes such as growth, differentiation, and migration on endothelial cells (Figure 1) [12,13,14,15,16,17].

**Figure 2:**
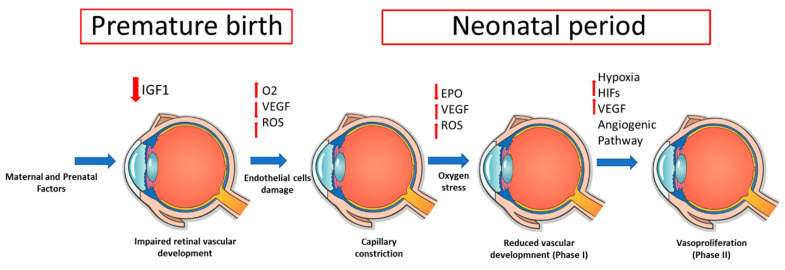
Description of the main known pathophysiological cascade of events leading to retinopathy of prematurity (ROP). Abbreviations: EPO, erythropoietin; HIFs, hypoxia-inducible factors; IGF-1, insulin-like growth factor 1; O2, oxygen; ROS, reactive oxygen species; VEGF, vascular endothelial growth factor.

From a biochemical point of view, ROP processes can be characterized by the following steps: (i) hypoxia-inducible factors (HIFs), which bind DNA to the hypoxia-responsive element and transcript angiogenic genes (i.e., VEGF, angiopoietins, erythropoietin) [13,17]. Hypoxia prevents HIFs from degradation by prolyl hydroxylases and, thus, allows them to translocate to the nucleus, causing angiogenic gene transcription. HIFs can be stabilized by oxidative compounds or inflammatory cytokines, mediated through nuclear factor kappa-light-chain-enhancer of activated B-cells (NFkB), which can lead to downstream angiogenic effector compounds [15,16,17]. (ii) Increased perinatal levels of prostaglandins (PGD2 and PGE2) and nitric oxide (NO) that cause the inability of the premature infant to control the oxygen requirement to the retina, modify vasomotor tone, and block the autoregulation [12,14,17]. (iii) Hypercapnia, which increases choroidal blood flow by a PGE2-dependent mechanism [12,14]. Hypercapnia also causes endothelial NO synthase (eNOS) activation, releasing NO, and mediates the delayed carbon dioxide-induced rise in ocular hemodynamics [14,16,17]. (iv) Low levels of IGF-1 because of retardation in retinal vessel growth, and (v) high levels of proinflammatory cytokines (Figure 2) [14,16]. Altogether, the aforementioned cascade of events contributes to endothelial toxicity, which, in turn, causes obliteration of the vessels.

In the perinatal period, the passage from an in-utero relative hypoxia environment to a relative hyperoxia one, in addition to oxygen support performed in DRs and in NICUs, has to be taken into consideration [12,13,14,15,16,17].

## 5. Oxidative Stress

Oxidative stress has been shown to be involved in both single and multiorgan failure of LBWs [18,19,20]. In the retina as well as in other central nervous system (CNS) areas, the lack of equilibrium between the production of pro-oxidants and the capability of the body to detoxify their harmful effects by anti-oxidants cause oxidative stress, involving, at this stage, the following known molecules: free radicals, NO, superoxide anion, and hydrogen peroxide [18,19,20,21].

In the perinatal period, especially at birth, the newborn is stressed by an oxidative challenge due to the fast change from a very-low intrauterine environment into a higher oxygen level one [19,20]. At this stage, the newborn is exposed to oxidative stress because of the low efficiency of antioxidant protection [18,19,20,21,22].

### 5.1. Main Pathophysiological Mechanisms and Mediators

#### 5.1.1. Hyperoxia/Hypoxia

Hyperoxia can be responsible for ROS generation by increasing superoxide due to the lack of a retina bloodstream autoregulation system that acts through a narrow range of perfusion pressure [20,21,22]. Hypoxia can increase ROS as well by slowing upstream events in the electron transport chain, and it raises the concentration of oxygen donors that, in turn, promote electron transfer to oxygen [21]. Hypoxia can also lead to the activation of NOS and nicotinamide adenine dinucleotide phosphate oxidase, enzymes that generate ROS and are involved in oxygen-induced retinopathy [20,21,22,23,24,25].

#### 5.1.2. Nitro-Oxidative Stress

Nitro-oxidative stress has an important role in microvascular degeneration, leading to ischemia in conditions such as ROP [20].

The synthesis of NO needs oxygen, which can have beneficial or harmful effects on the retina. The experimental model of oxygen-induced retinopathy (OIR) demonstrates that the expression and activity of endothelial NO increase under conditions of oxidative stress [26]. It is for this reason, NO reacts with reactive oxygen species, generating nitrite, nitrate, and peroxynitrite, which cause retinal microvascular damage, the so-called nitro oxidative stress [20,26,27]. Another pathophysiological mechanism involved in nitro-oxidative stress regards the local elevation of carbon dioxide (CO_2_), leading to impaired developmental and ischemic neovascularization. The mechanisms by which hypercapnia induces retinal microvascular degeneration preceding the proliferative preretinal neovascularization are still controversial and debated; however, in vitro hypercapnia has been shown to be a facilitator of nitration [28].

From a more detailed biochemical point of view, excess oxygenation results in the formation of ROS, which contributes to microvascular injury [28,29,30,31,32].

Several studies have pointed out a critical role for NO• in ensuing nitro-oxidative stress in ischemic retinopathies. The recently described markers of nitro-oxidative stress and major products of NO_2_•-mediated isomerization of arachidonic acid (AA), the transarachidonic acid (TAA) isomers, represent a new aspect of NO2-induced toxicity. Exposure of endothelial cells (ECs) to hypercapnia has been shown to inhibit their proliferation, migration, and differentiation into capillary-like structures. In preterm infants (PI), hypercapnia attenuates developmental retinal processes, impairs retinal angiogenesis, and contributes to the development of ROP. The mechanisms by which NO-derived reactive species participate in microvascular injury are not fully characterized. A novel peroxidation process mediated by NO_2_•, which results in cis- to TRANS-isomerization of AA, was recently described.

TAAs have recently been identified as mediators of nitrative stress in vivo, as they are released during nitrative stress and induce microvascular damage through an upregulation of TSP-1 in retinal microvascular ECs. Thrombospondin-1 (TSP-1) is an important protein synthesized and secreted by ECs, which inhibits angiogenesis by curtailing migration and inducing microvascular EC apoptosis. The recent identification of TAA-induced TSP-1 expression as an important pathway responsible for the ensuing microvascular injury provides a novel therapeutic target in these seriously debilitating disorders associated with nitrative stress [33,34,35].

#### 5.1.3. Lipid Peroxidation

Lipid peroxidation of cell membranes is due to inadequate oxygen tension [20]. The retina is highly sensitive to lipid peroxidation as it is composed of lipids with high levels of polyunsaturated fatty acids (PUFAs), such as docosahexaenoic acid (DHA), cis-arachidonic acid, and choline phosphoglyceride [20,29]. Nitric stress induces cis–trans isomerization of arachidonic acid, which causes vascular damage of the retina in a mouse model of ROP [29,30,31]. Plasma transarachidonic acid levels increase in microvascular damage [20]. In addition, through lipid peroxidation, platelet activation factor and lysophosphatidic acid are produced, which are known as proinflammatory and microvascular injury mediators [20].

## 6. Biomarkers

Based on the aforementioned findings, there is growing evidence that all metabolites and enzymes herein reported cannot be assessed in different biological fluids due to technological and laboratory limitations such as the short half-life of oxidative stress mediators and short-term result output. The issue is of clinical relevance in order to include biomarkers (BMs) in clinical guidelines.

In the last decades, there has been a growing interest in investigating the potential usefulness of BMs in the early detection of oxidative stress damage in newborns [19,36]. Recently, the Food and Drugs Administration (FDA), the European Medicine Agency (EMA), and, more recently, the National Institute of Health (NIH) established several statements for BM inclusion in perinatal clinical guidelines [37]. One of the main limitations refers to the possibility of BM assessment in different biological fluids, such as amniotic, urine, plasma, and cerebrospinal (CSF) fluids. In this respect, free radical measurement might be limited due to the different reactive and short-lived species, as well as to the possibility of their measurement in the so-called noninvasive biological fluids (urine, saliva) [19,37].

### 6.1. Malondialdehyde

Malondialdehyde (MDA) represents one of the final and highly toxic molecules of lipid peroxidation that is easily detectable in the retina [19]. It is a sensitive marker of oxidative stress that, interacting with DNA and proteins, may serve as a potentially mutagenic and atherogenic factor [18,38]. Although MDA constitutes a valuable marker of retinopathy in adults, under different conditions, in the perinatal period, there are only a few observations [38,39]. In this regard, Banjac et al. examined a cohort of 59 PIs, of whom 18 developed ROP and 41 did not, by assessing MDA blood levels at 33 GA. The authors found that the MDA levels were significantly increased in the ROP group. On the basis of the present findings, it has been speculated that MDA might be a promising BM of ROP in PIs [18,38,39].

### 6.2. 8-Hydroxy 2-Deoxyguanosine

It has been shown that when ROS causes strand breaks in DNA and base modifications, including the oxidation of guanine residues, its oxidation product, 8-hydroxy 2-deoxyguanosine (8-OHdG), can serve as a sensitive biomarker of oxidative DNA damage [40,41,42]. Recently, significantly increased plasmatic and urinary levels of 8-OHdG in a number of systemic diseases have been reported. Levels of products of oxidative DNA lesions in biological fluids (urine, serum, CSF) and tissues have been reported to be reliable biomarkers of oxidative stress [40,41,42,43,44].

Ates et al. have observed 8-OHdG in leukocyte DNA and urine samples of 50 patients with/without ROP. The authors found higher values in patients with ROP compared to patients without ROP. It has also been confirmed that there is a significant correlation between plasmatic levels of 8-OHdG in leukocyte DNA and plasmatic MDA and between levels of urine 8-OHdG excretion and urine MDA [40].

### 6.3. GSH/GSSG Ratio

The GSH/GSSG ratio is currently considered the most useful BM employed to assess oxidative stress [19]. GSH is a nonenzymatic antioxidant of cytoplasm; the GSH/GSSG ratio reflects the cytoplasmic redox status balance that is essential for cellular processes. The GSH/GSSG ratio can be measured with a method of high-performance liquid chromatography (HPLC) coupled to mass spectrometry (MS/MS) [19,36]. The characteristic of the measurement of GSH/GSSG depends on the fact that it can be studied on whole blood since GSH is within the blood cells and GSSG is found in plasma [19,36].

Ozieblo-Kupczyk et al., in a cohort of 30 PIs, investigated the correlation among the plasma concentration of antioxidant system parameters (i.e., superoxide dismutase (SOD), GSH, and GSH–GPX), measured at 1-7-14 days after birth, and the prevalence of ROP. The authors showed that SOD and GPX were significantly increased in PIs, with or without ROP, than in controls. Notably, lower GSH levels were observed in infants complicated by ROP. The results suggest that GSH could be a useful parameter to recognize early ROP development in PIs [45,46].

### 6.4. Protein Oxidation

Several amino acid residues can be modified by the action of ROS. Another biomarker in newborns that reflects oxidative stress is the oxidation of phenylalanine (Phe) residues in proteins [36]. It is known that neutrophils and macrophages produce hypochlorous acid in the presence of myeloperoxidase to attack bacteria. The reaction of hypochlorous acid with intermediate metabolites in the oxidation of phenylalanine leads to the production of 3-chlorotyrosine (3Cl-tyr), a useful marker of inflammation [34,36].

### 6.5. Lipids

The retina is susceptible to lipid oxidation; in fact, excessive oxygen tension causes lipid peroxidation, participating in the pathogenesis of ROP [36]. PUFAs are physiologically peroxidized by specific enzymes (lipoxygenase and cyclooxygenase), with the generation of prostaglandins, prostacyclin, thromboxane, leukotrienes, and lipoxins. In a pro-oxidant condition, PUFAs can be oxidized by free radicals, forming specific byproducts such as isoprostanes (IsoPs), isofurans (IsoFs), dihomo-IsoPs, neuroPs, and neuroFs [36]. These products can be measured with specific methods, such as HPLC–MS/MS and gas GS–MS/MS [36].

## 7. The Role of Scavenging Systems in ROP

Chemical molecules and enzymes, such as ascorbate, urate and reduced glutathione, and vitamin E (tocopherols and tocotrienols), prevent oxidation injury in biological membranes and lipoproteins [38]. Enzymatic antioxidants include catalase, SOD, and glutathione peroxidase (GPX). SOD enzymes show genetic polymorphisms that may be relevant in ROP [38]. They induce the dismutation of the superoxide radical into hydrogen peroxide and oxygen [38].

### 7.1. Glutathione

Hydrogen peroxide is reduced to water by GPX and catalases (CAT): it avoids conversion into the hydroxyl radical. In the presence of NADH and glutathione reductase, GSSG is reverted to GSH [38]. Recently, in LBWs (30–32 GA) at risk for ROP development, it has been shown that SOD and GPX blood concentrations were significantly increased than in those who did not develop ROP [47]. Notably, when low-risk cases were longitudinally monitored at term (40–42 GA), a significant decrease in SOD and GPX blood levels were observed. The authors suggested that high SOD and GPX levels, at earlier GA, are a compensatory attempt to counter the increased oxidant stress responsible for ROP [47]. Conversely, the progressive decrease in SOD and GXP can be suggestive of increased consumption of antioxidants in response to oxidative stress [38,47].

### 7.2. Nrf2/Keap1

The transcription factor nuclear factor erythroid-2 related factor 2 (Nrf2)/Kelch-like ECH-associated protein 1 (Keap1) is the regulator of antioxidant responses and it controls the up-regulation of other antioxidants such as hemeoxygenase-1 (HO-1) and NAD(P)H quinine oxidoreductase 1 (NQO1), enzymes implicated in glutathione biosynthesis (e.g., glutamate-cysteine ligase and glutathione synthase) [48,49,50,51]. The discovery of small molecules that upregulate Nrf2 responses in cell lines and tissues could represent a potential therapeutic pattern to reduce oxidative stress [49,50,51,52,53]. Indeed, an example of these new molecules is 2-cyano-3,12-dioxo-oleana-1,9-dien-28-oic acid (CDDO), a synthetic derivative of oleanolic acid. After many subsequent chemical modifications, CDDO resulted in the development of the triterpenoid dh404 (dihydro-CDDO-trifluoromethyl amide), which is able to enhance efficacy and reduce toxicity [48,54,55]. Although extremely promising, at this stage, no data is available yet on the Nrf2/Keap1 assessment in the biological fluids of PIs complicated by ROP.

### 7.3. Paraoxonase

Paraoxonase (PON) is another important factor involved in the antioxidant mechanism [18]. It is a Ca^2+^-dependent enzyme synthesized in the liver, which is found in many tissues and plasma. PON1 has an antioxidant function because it prevents the increase of reactive oxygen species quantity by hydrolyzing lipid peroxidation products [18]. It also protects cells from damage caused by oxidative stress. It was found that PON activity is higher in infants with ROP than in those without ROP. For this reason, Banjac et al. reported a positive correlation between oxidative molecule MDA and PON1, suggesting a PON1 protective action [18]. Hence, on the basis of the current data, it is supposed that PON1 could be a useful BM of ROP.

### 7.4. Stanniocalcin-1

Another molecule that seems to have a defensive effect against oxidative stress is stanniocalcin-1 (STC-1), which is a neuroprotective protein with anti-inflammatory and antioxidative stress properties [56]. STC-1 plays a role in the OIR stress response and development of pathologic vascular features in rodent OIR models by regulating VEGF levels [56]. STC-1 is a multifunctional protein that is upregulated by cellular stresses [57,58,59,60]. STC-1 protects neurons, photoreceptors, and retinal ganglion cells, and it also limits intraocular pressure, oxidative stress, and inflammation [61,62]. It seems that its neuroprotective effects are derived from uncoupling oxidative phosphorylation by induction of mitochondrial uncoupling protein-2. Therefore, it is hypothesized that STC-1 could have some anti-inflammatory and antioxidant effects underlying ROP [57,58,59,60]. Moreover, STC-1 regulates VEGF levels downstream of HIF1α. For these reasons, STC-1 expression is inducible by links between HIF1α and STC-1 promoters [63]. The present data suggest STC-1 modulates OIR severity, in part, due to its effect on VEGF production. The reduction of avascular and neovascular retinas in the presence of STC-1 might be associated with neuroprotective and anti-VEGF effects [56,63,64,65].

As for other BMs mentioned in this review, studies on the longitudinal assessment of STC-1 in different biological fluids are still in progress and eagerly awaited.

## 8. Nonenzymatic Scavenging Systems

Literature data on the effectiveness of different strategies on the prevention and treatment of ROP are still controversial and a matter of debate. This especially refers to vitamin E (VIT E), vitamin C (VIT C), omega 3, polyunsaturated fatty acids (PUFAs), and lutein administration as antioxidant agents that, at this stage, are the most investigated.

### 8.1. Vitamins E and C

VIT E is a free radical scavenger, capable of maintaining the integrity of the membrane in the cells of the retina by managing and reducing lipid peroxidation. In adult retinas, VIT E is highly concentrated, whilst in PIs, it is significantly decreased, up to 10% [66]. Literature data have shown the beneficial effects of VIT E supplementation on the incidence and severity of ROP [66]. The explanation may reside in its role of lipid-soluble antioxidant, which is able to prevent phase I ROP. In this regard, in animal models, VIT E treatment has been found to decrease ROP incidence in the absence of any side-effects [66]. However, the mechanism through which VIT E can be protective of the occurrence of ROP has not been fully elucidated. For example, it has been shown that polymorphisms in the cytochrome P450 4F2 can positively affect VIT E, suggesting its inclusion in clinical guidelines for ROP treatment. Therefore, further studies in an OIR model and in humans are awaited in order to validate VIT E’s neuroprotective action in PIs [66].

VIT C is the most potent concentration-dependent water-soluble antioxidant in the body and the principal antioxidant that quenches aqueous peroxyl radicals and lipid peroxidation products in plasma ex vivo [67]. It is a potent reducing agent and scavenger of free radicals in biological systems. VIT C is involved in the first line of antioxidant defense, protecting lipid membranes and proteins from oxidative damage. As a water-soluble molecule, it can work both inside and outside the cells and can neutralize free radicals and prevent free radical damage [67,68,69]. VIT C is an excellent source of electrons for free radicals that are seeking an electron to regain their stability. Indeed, it can donate electrons to free radicals and quench their reactivity [68,69]. In addition, it is effective in regenerating the antioxidant form of vitamin E by reducing tocopheroxyl radicals. This process protects membranes and other compartments of the cell from free radical-induced damage [67,68,69,70]. Generally, VIT C is oxidized to dehydroascorbic acid [67]. It may be converted back into ascorbic acid for reuse or may be metabolized as an anti-oxidant, neutralizing free radicals. In this case, dietary supplementation is needed in order to fulfill the vitamin’s requirement [67].

VIT C beneficial effects have been investigated in cell cultures and in humans [19]. In vitro, ascorbate is preferentially oxidized in plasma before other anti-oxidants (uric acid, tocopherols, and bilirubin), releasing electrons that neutralize and quench reactive species [19,20]. However, despite these promising anti-oxidant effects, in vivo VIT C results are still controversial and matter of debate, particularly on ROP prevention.

To the best of our knowledge, no data are available on changes in VIT C plasma concentrations that correlated with beneficial- or side-effects in PIs complicated by ROP. Indeed, there is evidence that VIT C crosses through the placenta from fetal (high) to maternal (low) bloodstreams by a gradient-of-concentration mechanism. Similarly, higher vitamin C levels in bone marrow have been found in PIs than in term infants [19,20].

### 8.2. Omega-3 and PUFA

In the last decade, increased interest has been shown for PUFA actions such as (i) modulators of processes affecting retinal health and disease [71,72], (ii) the prevention of ROP based on the role of long-chain PUFAs, a subgroup of PUFAs [73,74,75], and (iii) the role of diet supplementation with omega-3 (ω-3) PUFAs in the prevention and treatment of ROP [73,74,75,76,77,78,79].

From the biochemical point of view, two families of essential fatty acids exist in nature: ω-3 and ω-6 [71,72,73]. They contain a carboxyl head group and an even-numbered carbon chain, with at least two methylene-interrupted double bonds. These molecules are classified by the number of carbons, double bonds, and proximity of the first double bond to the methyl (omega) terminal of the fatty acid acyl chain. In this regard, the ω-3 family of fatty acids has a double bond at the third carbon, while the ω-6 family has a double bond at the sixth carbon [71,75]. Among different essential fatty acids, docosahexaenoic acid (DHA; C22: 6ω-3) is the main structural lipid located in the retina together with its substrate, eicosapentaenoic acid (EPA; C20: 5ω-3) [71,72,73,77,78].

Literature data suggest that ω-3 and ω-6 PUFAs, among different functions to date, are still matter worthy of investigation as they can exert beneficial effects on oxidative stress prevention [71,72,73]. In particular, (i) since they are present in the diet as protective vascular factors, they promote the formation of cytoprotective and anti-inflammatory metabolites [72,73,74,75], (ii) as substrates for phospholipase A, they determine the concentration of downstream pro-angiogenic or anti-angiogenic bioactive metabolites [72,76], (iii) they determine changes in the concentrations of bioactive lipid mediators synthesized during the retina’s response to stress stimuli [72,73,74,75,76], and (iv) DHA and AA, involved in a cascade angiogenic and pro-inflammatory events, are linked to transcription factors for genes that influence cell differentiation, metabolism, and growth [73,74,75]. Nonetheless, today, it is believed that the inflammatory and anti-inflammatory state of the tissue mainly depends on the lipid membrane composition [72]. On one side, pro-inflammatory 2-series prostaglandins and leukotrienes are produced through a ω-6 PUFA (AA)-mediated mechanism [72,73]. On the other side, anti-inflammatory neuroprotectins and D-series resolvins originate from the ω-3 PUFA DHA, whilst E-series resolvins and 3-series prostaglandins originate from EPA [76]. Altogether, several studies have been conducted in order to elucidate, in humans, the usefulness of ω-3 and ω-6 PUFAs in ROP prevention and treatment [73,76]. In this respect, Löfqvis et al. investigated whether the longitudinal assessment of cord and peripheral blood ω-3 and ω-6 LC–PUFA levels were correlated with the risk of ROP [76]. The authors conducted an open-label single-center RCT, comparing the effects of the parenteral lipid emulsion with (rich with ω-3 LC–PUFAs) or without fish oil (with olive oil-based clinoleic) on ROP. Cord and peripheral blood lipid plasma levels were measured at 1, 7, 14, and 28 days after birth and at 32, 36, and 40 postmenstrual GA. The results showed lower AA levels at 32 GA in PIs developing ROP. Receiving operating characteristic curve analysis reported that ω-6 LC–PUFA levels were predictors of ROP in the first month after birth [76]. Recently, Qawasmi et al., in their meta-analysis on 19 studies involving 1949 infants, assessed that PUFA supplementation enhanced visual acuity throughout the first year of life. In addition, supplementation of fish oil emulsion containing omega-3 and PUFAs in PIs has been shown to be associated with a low risk of ROP [71].

### 8.3. Lutein

Lutein (LT) is a member of the xanthophyll family of carotenoids mainly contained in dark green leafy vegetables, such as spinach and kale [80,81]. LT is a powerful antioxidant due to its unique chemical structure, which is not synthesized by humans. Therefore, its concentration in the human body is correlated to its dietary intake [82,83,84,85,86,87,88].

LT is characterized by having a hydroxyl group attached to each end of the molecule, making it more hydrophilic, thus reacting more strongly with singlet oxygen than other carotenoids [80,81].

The predominant LT site of concentration as a carotenoid is the central nervous system (CNS), specifically in the frontal and occipital cortex and hippocampus areas, which are of utmost relevance for learning and memory [80,81,87]. LT has also been shown to be specific in metabolic, energy, neurotransmission, and antioxidant pathways, suggesting its role in CNS development processes. Notably, LT selectively accumulates in the retina where it has been suggested to (i) enhance visual acuity by decreasing chromatic aberration [80,87], (ii) protect against light-induced oxidative damage by filtering the incident blue light on the retina [82,83], and (iii) scavenge reactive oxygen species generated in the retinal tissue [87]. Therefore, there is increasing interest in LT neuroprotective action, especially in retinal ischemia–reperfusion injury. Results in animal models and cell cultures reported that LT (i) prevents the increase of nitrotyrosine and PAR and, hence, apoptosis and cell loss in inner retinal neurons, (ii) protects retinal ganglion cells from H_2_O_2_-induced oxidative stress and cobalt chloride (CoCl^−2^)-induced hypoxia, (iii) preserves the retinal function, reducing the production of ROS, inactivating the NFκB signaling pathway, and decreasing the levels of oxidative markers, and (iv) prevents retina ERK activation, synaptophysin reduction, BDNF depletion, and subsequent neuronal loss [82,83,88].

### 8.4. LT and Biological Fluids

Among different LT sites of concentration, human milk appears particularly interesting in terms of the potential antioxidant factor [88]. The fact that higher levels of LT have been found in both colostrum (>140 μg/L) and in mature human milk (2–3 times more) than other carotenoids suggests the relevance of an appropriate maternal daily diet intake [88,89]. Nevertheless, it has to be pointed out that human milk constitutes the best and only dietary resource of LT for babies in the first months of life. The finding is of note, bearing in mind that milk-formulae do not contain LT and its metabolites affect the potential anti-oxidant action of feeding in healthy and sick infants [88,89,90]. In this regard, FDA has approved the dietary supplementation of LT and its metabolites (i.e., zeaxanthin) as potential protective agents against photo-oxidation, photodestruction, and physiological visual function achievement, hence, having a shutdown effect on oxygen [80,81,82,88].

Although there have been promising results in animal models and cell cultures, in humans, the actions of LT and its metabolites are still controversial and debated. On the one hand, the LT pattern of concentration in human milk is suggestive of the need to restore physiology when human milk is not available, while on the other hand, RCTs’ results argue against this hypothesis [88,89,90]. This especially refers to recent RCT studies by Dani et al. and Romagnoli et al., who recruited *n* = 114 and *n* = 63 PIs, aged less than <32 GA, respectively, who were supplemented by LT or placebo. In both observations, the authors concluded that LT and its metabolites were ineffective in ROP prevention and outcome [85,86]. The results opened the view to further studies aimed at investigating LT administration modalities, dose and length of the treatment, as well as the need for reference curves of LT in biological fluids of healthy PIs [85,86]. The latter issue is strategical in order to establish the effective LT need in PIs complicated by ROP.

Recently, it has been shown that fetal/neonatal LT and 3’-oxolutein blood levels in PIs and term infants are GA-, gender-, and delivery mode-dependent. In particular, higher LT and 3’-oxolutein levels were observed more in PIs than in term infants, as well as in preterm and term females than males. Conversely, lower LT and 3’-oxolutein blood levels were found in PIs and term infants born by cesarean section [82].

Despite, LT’s predominant site of concentration in CNS, its neurotrophic role still needs to be elucidated. In this regard, Picone et al. conducted an observational study in healthy PIs (*n* = 50) and term healthy infants (*n* = 82), with GA between 33 and 42 weeks, correlating LT cord blood levels with the concentrations of a well-established neurobiomarker, namely, activin A (act A). Briefly, act A is a growth factor consisting of two beta subunits. Its receptors and binding proteins are located in CNS. Act A has a role in response to acute neuronal damage and in neuroprotection [83]. High levels of act A in the biological fluids (cerebrospinal, cord, blood, urine) have been found in both fetuses and newborns complicated by hypoxia, perinatal asphyxia, and cerebral hemorrhage [83]. Results showed that LT and act A were GA- and gender-dependent in both PIs and term infants, with higher levels in females in the III-trimester of gestation. In fact, the pattern of concentration of LT and act A was characterized by higher levels at 33–36 GA, followed by a progressive decrease from 37 GA until a dip at term. The results offered further information on the debated role of LT as a CNS trophic factor.

## 9. Discussion

ROP is the leading cause of potentially preventable blindness in children [4,7]. Major risk factors of ROP include GA, BW, oxygen supply, hypercapnia, maternal and newborn pathologies, and NICU therapeutic strategies [1,2,5]. The present multifactorial pattern contributes to compromise physiological vascular growth in the neonatal retina, such as an involution of the central choroid and choriocapillaris and photoreceptor injury leading to altered visual detection in the last stages [1,2,3].

Oxidative stress has been described as the major molecular mechanism involved in the pathogenesis of ROP [18,21,38]. Therefore, in the last few years, there has been an increasing interest in identifying early BMs of ROP in order to better understand the pathogenic mechanisms and its prevention strategies. Since ROP can be considered as the single or partial expression of CNS damage in PIs, there is the possibility that brain-specific BMs, such as oxidative stress markers, could be employed for ROP detection. As for CNS damage, we argue that it is mandatory that BMs for ROP are to be validated by the same criteria adopted by FDA, EMA, and NIH for CNS damage (Table 1). According to the statements officially proposed by those institutions, we offered an update on the most recent oxidant and antioxidant BMs.

In this setting, before any conclusion can be drawn on a panel of BM pros and cons, it is necessary to focus on the predetermined criteria requested for a BM before inclusion in clinical guidelines. In detail, the “optimal” BM needs to fulfill the following criteria: (i) alternative and direct indicator of damage, (ii) early predictor of the degree and location of the injury, (iii) indicator of the extent of the lesion, monitoring the progression of the disease, (iv) well studied in the pediatric population, (v) measurable by available commercially kits worldwide, with good reproducibility, (vi) presence of reference range for the pediatric population, and (vii) assessment in different biological fluids [37].

In the last decade, a panel of BMs has been investigated and measured in biological fluids, in both animal models and in humans, in order to evaluate which is the best early predictor of ROP and lesion extension. Literature data show that among a panel of BMs currently investigated, MDA, GSH/GSSG, 8-Hdhg, and PUFAs appear to be the most promising for the diagnosis and treatment monitoring of ROP [18]. The findings are not surprising since they are involved partly or mainly in the well-known cascade of events leading to oxidative stress damage. Thus, their potential role in the prevention of retinal damages and irreversible vision disorders is justified (Table 1) [12,17,36].

To date, no conclusive clinical protocols or guidelines regarding ROP diagnosis and treatment in the neonatal period involving BMs have been approved. The main explanation resides in the fact that, at this stage, no BM has been validated by the correlation with the so-called standard of care procedures, such as fundus oculi and retinal angiofluorography patterns [3,9]. The finding is noteworthy, bearing in mind that these procedures are the only ones able to provide useful information on the severity and extension of ROP lesions, as well as on the effectiveness of the therapeutic strategies performed.

The majority of the BMs investigated were shown (i) to be valuable indicators of the occurrence of retina damage, (ii) to be trustworthy indicators of the severity and extension of the lesions except for GSH/GSSG (the latter requires further investigations in order to elucidate the present issues), (iii) to have a weakness in the possibility of longitudinal monitoring (GSH/GSSG, Nrf2/Keap1, PON1) and also in the lack of reference values in the different biological fluids at the age of investigation.

The laboratory measurement techniques and subsequent result output for each BM warrant further discussion. The main limitations reside in the different techniques currently adopted in terms of reproducibility, and the standardized measurement procedures in different biological fluids, particularly urine and saliva, are of utmost importance for PI monitoring. This especially holds for HPLC, ELISA, RT, and qPCR, which, at this stage, are still not certified for BM measurement in non-invasive biological fluids. Further improvements in the assessment techniques and manufacturers’ production are needed in order to offer physicians a trustable BM result output that is independent of the measurement procedure. In this regard, the shortest timing for result output (less than 2–4 h) is requested in order to perform the therapeutic strategies at the due time.

Finally, further limitations reside in the design of the trials themselves in terms of a lack of multicenter investigations in the wider population to assess a single or a panel of BMs.

Last but not least, the anti-oxidant action of the nutritional component has to be taken into due account. There is evidence that vitamins, specific lipids, dietary elements, and adequate caloric intake play an important role in ROP prevention [88,90,91]. Literature data have shown that in optimizing nutritional support (i.e., lipids, total calories) and weight gain, there is a decay in severe ROP incidence in PIs, thus contributing to normal retinal vascularization. This especially holds for IGF-1, ω-3 long-chain PUFAs that have been involved, under a still unknown mechanism, in the suppression of the inflammatory response in the antiangiogenic and neuroprotective mechanisms of the retina [91]. Notably, poor postnatal weight gain has been related to the occurrence of severe ROP. In fact, by means of specific algorithms, it has been shown that enhancing nutritional intake and weight gain limits ROP occurrence in PIs. [91]. Moreover, it has been shown that vitamin supplementation, especially VIT E and VIT C, through their antioxidant effect, contributes to prevent and reduce retinal damage [19,20]. More recently, LT, a natural antioxidant at high concentrations in maternal diet and human milk, has been suggested to play a protective role against photo-oxidation and lipid peroxidation in the retinal tissue [90]. Although LT has a high concentration in macula densa and eyes, supporting the hypothesis of its anti-oxidant action, at this stage, there is no strong evidence about LT and its role in ROP prevention [19,20]. In particular, for LT, there are still no conclusive data on the dose and the method of administration for therapeutic purposes [19,20]. Further studies in the wider population are therefore requested.

## 10. Future Perspectives

In the present series, we have highlighted the pros and cons in terms of the potential inclusion in clinical practice of BMs as predictors and indicators of therapeutic strategies in PIs complicated by ROP.

Future perspectives will regard the need for multicenter studies aimed at fulfilling the criteria requested by institutions for BM inclusion in ROP prevention and treatment clinical guidelines. Of course, at present, new and promising results can be obtained, thanks to technological improvement. This refers to the metabolomics approach that offers a holistic metabolite pattern to detect new metabolites and elucidate the still-unknown pathophysiological steps leading to ROP [19,92,93,94].

Future studies will also regard the evaluation through BM assessment in different biological fluids of the effectiveness of the therapeutic strategies performed, as well as the early detection of NICU treatment side-effects. In this regard, LT, vitamins, and, more recently, caffeine and melatonin will constitute the bulk of new investigations open to a close relationship among BMs and consolidated and new therapeutic strategies for PIs at risk for ROP [20,95].

## Figures and Tables

**Table 1:** Optimality items for a BM according to FDA and EMA criteria.

BM	RD	DI	LE	LM	Ak	RC	BF	Ref
MDA	Y	Y	Y	Y	SP, HPLC	N	B	[18,38,39]
8-OHdG	Y	Y	Y	Y	HPLC	N	U, B	[40,41,42,43,44]
GSH/GSSG	Y	N	N	Y	HPLC, MS/MS	N	B	[19,36,45,46]
SOD	Y	Y	Y	Y	HPLC	N	B	[38,47]
GPX	Y	Y	Y	Y	SP	N	B	[38,47]
Nrf2/Keap1	Y	N	N	Y	RT-PCR, ELISA	N	B	[48,49,50,51,52,53,54,55]
PON1	Y	Y	Y	Y	SP	N	B	[18]
STC-1	Y	Y	Y	Y	qPCR, ELISA	N	B	[56,57,58,59,60,61,62,63,64,65]

Abbreviations: BM, biomarker; FDA, Food and Drugs Administration; EMA, European Medicine Agency; RD, retina damage marker; Y, DI, degree of injury; LE, lesion extension; LM, longitudinal monitoring; Ak, available kit; RC, reference curve; BF; biological fluid; ref, references; yes; N, no; MDA, malondialdehyde; 8-OHdG, 8- hydroxy 2-deoxyguanosine; GSH/GSSG, glutathione /glutathione disulfide; SOD, superoxide dismutase; GPX, glutathione peroxidase; Nrf2/Keap, nuclear factor erythroid-2 related factor 2/Kelch-like ECH-associated protein 1; PON1, paraoxonase; STC-1, Stanniocalcin-1; SP, spectrophotometry; HPLC, High-performance liquid chromatography; MS/MS, tandem mass spectrometry; RT-PCR, reverse transcription polymerase chain reaction; ELISA, enzyme-linked immunosorbent assay; qPCR, quantitative polymerase chain reaction; B, blood; U, urine.
